# Safflower ( *Carthamus tinctorius* L.) Seed Oils: Effect of Extraction Process and Cultivars on Chemical Composition, Physicochemical Parameters and Nutritional Quality Index

**DOI:** 10.1002/fsn3.71954

**Published:** 2026-06-04

**Authors:** Chaimae El Kourchi, Oumayma Belhoussaine, Rim Mohammed Ali, Mohammed Amakhmakh, Khalid M. Sumaily, Agnese Santanatoglia, Filippo Maggi, Giovanni Caprioli, Abdelhakim Bouyahya, Mohamed Tabyaouı, Hicham Harhar

**Affiliations:** ^1^ Department of Chemistry, Faculty of Sciences Mohammed V University in Rabat Rabat Morocco; ^2^ Laboratory of Applied Organic Chemistry Faculty of Sciences and Techniques Sidi Mohamed Ben Abdellah University Fez Morocco; ^3^ Clinical Biochemistry Unit, Pathology Department, College of Medicine King Saud University Riyadh Saudi Arabia; ^4^ Chemistry Interdisciplinary Project (ChIP), School of Pharmacy University of Camerino Camerino Macerata Italy; ^5^ Laboratory of Human Pathologies Biology, Faculty of Sciences Mohammed V University in Rabat Rabat Morocco

**Keywords:** extraction process, fatty acid, nutritional properties, safflower, seed oil, tocopherols

## Abstract

This paper examines the nutritional and metabolic characteristics of *Carthamus tinctorius* seed oils produced in two regions across Morocco and assesses how various extraction methods, Soxhlet, cold pressing, and maceration, affect oil yield and the presence of bioactive compounds, which establishes the data on Moroccan cultivars that have limited information. Soxhlet extraction provided the highest oil yield, with the oil being rich in linoleic acid (40.45% ± 0.17%–80.25% ± 0.16%), a polyunsaturated fatty acid. Despite the high PUFA content raising concerns about oxidative stability, both varieties extracted without solvent had peroxide value (9.62 ± 0.03 and 9.45 ± 0.04 meq O_2_/kg) below the recommended limits for cold pressed oils, and high levels of tocopherol, especially α‐tocopherol (580.66 ± 0.27–837.61 ± 0.22 mg/kg), contribute to antioxidative protection. Also, the key nutritional indicators have highlighted this oil's health benefits. These fundings suggest that this oil is a promising source of dietary lipids, exhibiting excellent values for multiple nutritional indices, including the h/H ratio (3.50 ± 0.06–13.53 ± 0.013), the atherogenicity index (0.079 ± 0.002–0.39 ± 0.015), and the thrombogenicity index (0.211 ± 0.001–0.639 ± 0.01), suggesting potential advantages for cardiovascular health. This research contributes to determining the most appropriate production method for obtaining safflower oil with desirable quality and nutritional properties, thereby paving the way for the potential use of these oils in the agri‐food, cosmetic, and pharmaceutic sectors.

## Graphical abstract

*M. pulegium* essential oil study emphasizes molecular structure analysis and bioactivity. Chemical composition varies based on geographical origin. In silico analyses confirm binding affinities. Essential oil shows potent insecticidal activity with LD_50_ and LT_50_.

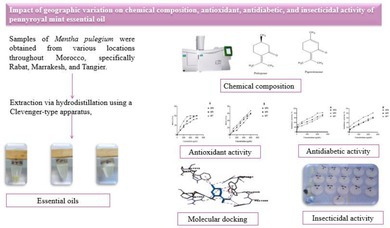

## 1 Introduction

The contemporary world is confronted with significant challenges, including the necessity to enhance productivity while concomitantly reducing environmental impact, adapting to climate change, and ensuring the availability of renewable energy alternatives (Ortiz et al. 2020). The increasing demand for food, alongside the growth of the human population, requires the intensification of agricultural practices to maximize food production and reduce the risk of famine and epidemics (El‐Hamidi and Zaher 2018). Consequently, food production must not only meet dietary needs but also promote health. The mounting evidence suggests that a diet consisting primarily of plant‐based diets may hold the potential to reduce the risk of developing conditions such as diabetes, cardiovascular diseases, hypertension, obesity, and metabolic syndrome (Lonnie and Johnstone 2020; El Kourchi, Belhoussaine, Harhar, et al. 2024).

In the realm of food products, oilseeds occupy a prominent position, ranking fourth among major food items, following cereals, vegetables, and melons. The demand for oilseed crops is on the rise, driven by several factors, including population growth, evolving dietary preferences, and the increasing demand for renewable bioproducts (Ahmad et al. 2021). A wide array of plants is utilized in the production of vegetable oils, derived from seeds, pulp, or feathers. These oils play a pivotal role in human activity, serving as a crucial energy source. The applications of these oils are diverse, extending beyond culinary uses to include cosmetics and food supplements. It is therefore essential that cooking oils are safe for consumption, as they are integral to the human diet. Due to the growing demand for vegetable oils and environmental pollution, a robust quality control system across the entire vegetable oil production chain–from cultivation to storage and marketing–is imperative (Zhou, Zhao, et al. 2020). Vegetable oils are primarily composed of triacylglycerols, which are esterified with fatty acids (FAs) that vary depending on the plant source (Verhé et al. 2006). FAs are classified into saturated (SFAs), monounsaturated (MUFAs), and polyunsaturated (PUFAs) based on their degree of saturation. Additionally, vegetable oils contain unsaponifiable components like polyphenols, phytosterols, minerals, and fat‐soluble vitamins (Ganesan et al. 2018). PUFAs, such as linoleic acid (C18:2 n‐6) and linolenic acid (C18:3 n‐3), are essential fatty acids (EFAs) due to their role in modulating lipids and reducing cardiovascular disease (Simopoulos 2016; Vera‐Candioti et al. 2021). However, PUFAs are prone to oxidation, which negatively impacts oil quality and leads to undesirable flavors. The oxidation process is influenced by factors such as unsaturation levels, pro‐oxidants (e.g., chlorophylls), and the presence of antioxidants (e.g., tocopherols), along with external factors like light, temperature, and oxygen exposure (Fadda et al. 2022). Addressing these challenges requires optimization of extraction methods to preserve oil quality during processing and storage (Grosshagauer et al. 2019). A wide range of extraction methods are employed in the production of vegetable oils, encompassing both conventional technologies and contemporary techniques (Ivanovs and Blumberga 2017). The choice of extraction method and solvent is critical in determining oil quality (Santos et al. 2013). Soxhlet extraction yields the highest oil content, but the associated high temperature and extended extraction time may degrade oil quality. Mechanical pressing preserves more bioactive compounds, though it is less efficient in terms of yield. Maceration, while slow, offers another extraction approach.

Safflower (*Carthamus tinctorius L*.), a xerophilic plant of the Asteraceae family, is well‐adapted to arid and semi‐arid climates (Abou Chehade et al. 2022). It is cultivated for its oil and dye, with major producers including India, United States and Ethiopia (Liu et al. 2016). The oil is then utilized by various industrial sectors, including cosmetics, pharmaceuticals, food and feed production (Ergönül and Özbek 2020). The composition and quality of safflower oil can vary depending on the biotope, climate, harvest method and genetic factors (Tonguc et al. 2023). The oil is rich in oleic and linoleic acids, which have been linked to numerous health benefits, including anti‐inflammatory, vasodilator, and neuroprotective effects (Delshad et al. 2018). The oil finds application within traditional medicine in Iran as a cure of liver and cardiac conditions, whilst in India it is employed for the treatment of rheumatism and sores (Eneogwe et al. 2026). The fatty acid composition of safflower oils is subject to variation according to cultivar and extraction method, with different quantities of oleic acid and linoleic acid being present. However, traditional methods such as soxhlet and maceration are limited by factors such as the necessity for protracted extraction times, excessive solvent consumption and dependence on water and electricity. Consequently, alternative processes are continually being studied to improve these conditions, with a view to achieving high, fast and clean yields.

This study aims to explore the impact of environmental biotope and extraction methods on the lipid profile of *Carthamus tinctorius* seed oils by evaluating their physicochemical properties, nutritional indices, and functional aspects.

## 2 Materials and Methods

### 2.1 Chemicals

Various chemicals were employed as analytical reagents, including ethanol, chloroform and acetic acid, all sourced from Professional Lab (Casablanca, Morocco). High‐performance liquid chromatography (HPLC grade) isopropanol and isooctane were used as mobile phases during analyses. Additionally, α‐tocopherol (purity: 96%) was used as a standard, obtained from Sigma Aldrich Co. (St. Louis, MO, USA). Each of these chemicals was crucial for ensuring the precision and accuracy of the experimental procedures.

### 2.2 Botanical Matter

Two safflower cultivars seeds belonging to the same variety and cultivated in two distinct areas in Morocco were utilized in this study. The first cultivar was harvested from the Souss‐Massa region in Ait melloul (30°20′03″ N, 9°29′50″ W), while the second came from the Guelmim‐Oued Noun region in Guelmim (28°59′17″ N, 10°03′27″ W). All samples were gathered at full maturity. Prior to analysis, the samples were thoroughly cleaned, sorted, and inspected, with any foreign particles or damaged seeds being removed to ensure the integrity of the material for further examination.

### 2.3 Proximal Analysis

Proximal analysis was conducted following ISO standardized methods. Moisture content was determined using the ISO 662:2016 method, where samples were dried at 105°C until constant weight was achieved. Ash content was measured according to ISO 18122:2015, with samples incinerated in a muffle furnace at 500°C for 4 h until white ash formed. Fat extraction followed ISO 659:2009, utilizing a Soxhlet apparatus for 6 h. The results for moisture, ash, and fat content were expressed as percentages, ensuring accurate representation of the proximate composition.

### 2.4 Extraction Protocol

Safflower seeds from different regions underwent various extraction methods: solvent‐free cold pressing and solvent extraction using both cold maceration and Soxhlet techniques.

*Mechanical extraction*: The cold pressing of the safflower seed was carried out using Komet DD 85 G presses (IBG Monforts Oekotec GmbH, Mönchengladbach, Germany) at a temperature of 90°C. This temperature refers to a heater that comes into contact with the extractor tube, with the purpose of applying heat to the sample in order to facilitate the extraction procedure. The extracted oil was filtered, separated from the paste, and transferred to brown glass bottles for storage at 4°C (El Bernoussi et al. 2020).

A critical step in the solvent extraction process involved grinding the seeds into a fine powder. For this purpose, the use of a Deutschmann DM‐250G 1800 W electric grain mill proved effective and efficient.

*Soxhlet extraction*: 40 g of seed powder was extracted with 260 mL of petroleum ether for 6 h. The solvent was evaporated using a rotary evaporator at 50°C under reduced pressure. The oil was then stored in brown bottles at 4°C (Boujemaa et al. 2020).

*Maceration*: In this study, extraction by maceration was carried out as described by Eddahhaoui et al. (2022) with a few modifications. The safflower seed powder (40 g) was stirred with 260 mL of petroleum ether for 48 h at room temperature. The solvent was removed by rotary evaporation at 50°C, and the oils were stored in brown glass bottles at 4°C.

### 2.5 Fatty Acid Content

Fatty acid methyl esters (FAMEs) were prepared and analyzed using flame ionization detection (FID) coupled with a Varian CP‐3800 gas chromatograph (GC) equipped with a CP‐Wax 52CB column (30 m × 0.25 mm). The column temperature was initially set at 170°C, ramped to a final temperature of 230°C at a rate of 4°C per minute. Helium was used as the carrier gas at a flow rate of 1 mL/min. Data were processed using Varian Star Workstation v 6.30, and the fatty acid composition was expressed as a relative percentage (Gharby et al. 2021).

### 2.6 Qualitative, Nutritional, and Metabolic Fatty Acid Indexes

The fatty acid composition of safflower oils was used to calculate several qualitative indices. These included the content of saturated (SFA), unsaturated (UFA), monounsaturated (MUFA), and polyunsaturated (PUFA) fatty acids, as well as the PUFA/SFA ratio and the unsaturation index (UI), following the method of Haugaard et al. (2006), Mutlucan and Önder (2025). Additionally, the oxidizability (Cox), oxidative stability (OS), and peroxidability index (PI) were determined using the formulas provided by Plaha et al. (2023), Moknatjou et al. (2015).

(1) $$\frac{\text{PUFA}}{\mathrm{SFA}}=\frac{\sum \text{Polyunsaturated}\;\mathrm{FA}}{\sum \text{Saturated}\;\mathrm{FA}}$$

(2) $$\begin{array}{c} \mathrm{UI}=\left(\text{monoenoic}\right)+\left(2\times \text{dienoic}\right)\\ \;+\left(3\times \text{trienoic}\right)+\left(4\times \text{tetraenoic}\right)\\ \;+\left(5\times \text{pentaenoic}\right)+\left(6\times \text{hexaenoic}\right) \end{array}$$

(3) $$\mathrm{Cox}=\frac{\left[C18:1+\left(10.3\times C18:2\right)+\left(21.6\times C18:3\right)\right]}{100}$$

(4) $$\mathrm{OS}=\text{MUFA}+\left(45\times C18:1\right)+\left(100\times C18:3\right)$$

(5) $$\mathrm{PI}=\left(0.025\times C18:1\right)+C18:2+\left(2\times C18:3\right)$$

The Nutritional value index (NVI), atherogenicity index (AI), thrombogenicity index (TI), hypocholesterolemic:Hypercholesterolemic (h:H) Ratio, and Health‐Promoting Index (HPI) were calculated as lipid nutritive indices in accordance with Chen and Liu (2020), Dal Bosco et al. (2022).

(6) $$\mathrm{NVI}=\frac{C18:0+C18:1}{C16:0}$$

(7) $$\mathrm{AI}=\frac{C12:0+\left(4\times C14:0\right)+C16:0}{\mathrm{UFA}}$$

(8) $$\mathrm{TI}=\frac{C14:0+C16:0+C18:0}{\left(0.5\times \text{MUFA}\right)+\left(05\times n6\text{PUFA}\right)+\left(3\times n3\text{PUFA}\right)+\left(\frac{n3\text{PUFA}}{n6\text{PUFA}}\right)}$$

(9) $$h:H=\frac{C18:1+\sum \text{PUFA}}{C12:0+C14:0+C16:0}$$

(10) $$\mathrm{HPI}=\frac{\mathrm{UFA}}{C12:0+\left(4\times C14:0\right)+C16:0}$$

Finally, using an enzyme approach called the “products/substrate ratio,” a variety of indices were used for metabolic indicators in order to quantify the activity of desaturases and elongase (Dal Bosco et al. 2022; Kurt 2018).

(11) $$\text{elongase}=\frac{C18:0}{C16:0}\times 100$$

(12) $$\text{Desaturase}=\frac{C18:1}{C18:0+C18:1}\times 100$$

(13) $$\text{Oleic desaturation}=\frac{C18:2+C18:3}{C18:1+C18:2+C18:3}\times 100$$

(14) $$\text{Linoleic desaturation}=\frac{C18:3}{C18:2+C18:3}\times 100$$

### 2.7 Physicochemical Parameters

The free fatty acid (FFA) content, expressed as a percentage of oleic acid, was measured alongside specific extinction coefficients (K_232_ and K_270_), peroxide value (PV) and iodine value (IV), following AOCS methods Ca 5a‐40, Ch 5‐91, Cd 8b‐90 and Cd 1c‐85, respectively. Chlorophyll and carotenoid pigment contents were analyzed using a UV spectrophotometer at wavelengths of 670 and 470 nm, corresponding to pheophytin and lutein fractions, respectively, as described by Belhoussaine et al. (2024).

### 2.8 Tocopherols Content

Tocopherol composition was analyzed using high‐performance liquid chromatography (HPLC) following ISO 9936: 2016 standards. The analysis was performed on a Shimadzu system equipped with a fluorescence detector and LabSolutions integration software. An autosampler injected 0.25 g of an oil solution onto a 25 cm diol column (4 mm internal diameter, 5 μm film thickness). The mobile phase was a mixture of isopropanol/isooctane (1:99, v/v), with a flow rate of 1.2 mL/min. Detection wavelengths were set at 290 nm (excitation) and 330 nm (emission). Tocopherols were quantified using external α‐tocopherol standards (Idrissi et al. 2022).

### 2.9 Statistical Analysis

All measurements were performed in triplicate, and data were analyzed using GraphPad Prism version 8.4.2. The results in tables and figures are expressed as mean ± standard deviation (SD), derived from three independent experiments. Statistical differences (*p* < 0.05) between the safflower varieties and extraction methods were evaluated using Tukey's test. Pearson's correlation coefficient was employed to examine the relationships between the various parameters analyzed in this study.

## 3 Results and Discussion

### 3.1 Proximate Analysis

A food's proximate composition, including moisture, ash, fat, protein and carbohydrate content, is vital for product development, quality control and regulatory compliance. As illustrated in Table 1, significant differences are evident between safflower seeds from two different localities. In the context of oilseeds storage, it is imperative to exercise meticulous attention to their water content. This preventive measure is implemented to avert the risk of oxidation, a process that can alter the nutritional and organoleptic properties of the seeds. The presence of water can encourage the proliferation of contaminating micro‐organisms (Kahyaoglu and Kaya 2006). In the present study, the moisture content of safflower seeds cultivated in the Ait Melloul region (SA) was found to be higher than that of seeds from Guelmim (SG), with a percentage difference of 1.84%. The moisture content reported by Yu et al. (2013) for Korean localities (6.9%–8.1%) aligns with the findings of this study. It is noteworthy that the observed water content in this study exceeds the levels documented in previous research (Yu et al. 2013; Shirvani et al. 2016). Consequently, this moisture content is consistent with the optimal range for the majority of seed species, which typically ranges from 6.3% to 11.3% by weight (Martínez et al. 2017). In addition to moisture, ash content refers to the inorganic residue that remains following the extraction of water and organic compounds through the application of heat in the presence of oxidizing agents. This method provides a quantitative assessment of the total mineral content present in a given food sample (Oubannin et al. 2022). This content also showed significant variation (*p* < 0.05) between the two varieties. Ash content ranged from 1.57% (SG) to 3.86% (SA). These values are comparable to the mineral content reported by Mansouri et al. (2018) for Moroccan safflower varieties (3.11%–3.60%) and by Yu et al. (2013) for Korean varieties (2.7%–3.3%), though our findings for Guelmim seeds were lower. It was observed that seeds with a high oleic acid content exhibited ash contents ranging from 2.68% to 4.87% (Kolláthová et al. 2019). This range of variance is consistent with that observed in SA seeds. This finding suggests a potential correlation between proximal seed composition and both environmental and genetic factors, as well as lipid profile.

**TABLE 1:** Proximate composition (%) of safflower seeds from Ait Melloul and Guelmim regions. Data are presented as mean ± SD.

	SA	SG
Dry matter	91.345 ± 0.134^a^	93.188 ± 0.088^b^
Moisture	8.655 ± 0.134^a^	6.813 ± 0.088^b^
Ash	3.860 ± 0.226^a^	1.575 ± 0.247^b^
Total fat	28.675 ± 0.672^a^	26.325 ± 0.629^b^

*Note:* The values of compounds are expressed as mean ± standard deviation. Values within each row with different superscript letters (a–b) are significantly different at *p* ≤ 0.05.

Abbreviations: SA, Safflower of Ait melloul; SG, Safflower of Guelmim.

### 3.2 Oil Yields and Fatty Acid Profile

As indicated in Table 2, the oil yields and fatty acid content of the two safflower seed varieties studied are expressed in percentages. A significant difference was observed in these values depending on the cultivar and the extraction method. Geographically, the seeds from Ait melloul exhibited a higher oil yield content than those from Guelmim. Liersch et al. (2020) reported that environmental factors play a dominant role, contributing 56.41% to oil yield variation, while genotype accounted for only 15.50%. Moreover Tonguc et al. (2023) reported a higher amount of oil content when compared to our study. This was between 29.17% and 34.53% of 13 safflower genotypes. Regarding extraction methods, Soxhlet extraction provided the highest oil yield for both cultivars, with 28.67% and 26.32% for SAS (Ait melloul safflower oil extracted by Soxhlet) and SGS (Guelmim safflower oil extracted by Soxhlet), respectively. Mechanical extraction yielded 25.3% and 22.55% for SAP (Ait melloul safflower oil extracted by cold press) and SGP (Guelmim safflower oil extracted by cold press), respectively, while cold maceration yielded the lowest amounts, 15.77% and 14.65% (SAM and SGM, respectively). Seeds from Ait melloul consistently produced more oil, regardless of the extraction method. Soxhlet extraction, while yielding the most oil, also causes heat‐driven molecular bond disruption and oil release, making it the most efficient (Zhang et al. 2022). Mechanical pressing, on the other hand, can lead to a 25% oil loss compared to solvent extraction methods (Matthäus 2012). The findings, as established by the results of the study, demonstrate that temperature and extraction time are two critical factors impacting vegetable oil yield. In this context, soxhlet extraction employs a combination of heat and a relatively brief extraction time. Conversely, maceration necessitates a protracted extraction period to ensure the successful production of oil (El Bernoussi et al. 2020). The yield differences between extraction methods, as well as genetic and environmental factors, are crucial in determining the oil yield of safflower seeds. It is evident from extant literature that yield variability can be ascribed to myriad factors, including genetic characteristics, environmental factors, cultivation methodologies, harvesting time, storage time, and the extraction process (Ramos‐Escudero et al. 2019). Moreover, Tonguç et al. (2023) have also indicated that when rhizobacteria were applied in unfertilized and rain‐fed growing conditions, it enhanced 1000‐seed weight, and oil yields. Furthermore, Salaberría et al. (2016) reported that seeds rich in oleic acid tend to produce the highest oil yield. However, in this study, the oil contained low oleic acid levels, ranging from 9.04% to 23.09%.

**TABLE 2:** Oil yield and fatty acid (%) composition of safflower seeds from Ait Melloul and Guelmim using different extraction methods.

	SAP	SGP	SAS	SGS	SAM	SGM
Oil yields %	25.3 ± 1.20^a^	22.55 ± 1.06^b^	28.68 ± 0.67^c^	26.33 ± 0.63^a^	15.78 ± 0.74^d^	14.65 ± 0.28^e^
Myristic acid	0.15 ± 0.01^a^	0.17 ± 0.01^a^	0.13 ± 0.00^a^	0.19 ± 0.01^a^	2.83 ± 0.22^b^	2.13 ± 0.11^c^
Palmitic acid	6.96 ± 0.24^a^	6.80 ± 0.00^a^	6.94 ± 0.04^a^	6.42 ± 0.01^b^	15.76 ± 0.11^c^	15.33 ± 0.02^d^
Margaric acid	0.05 ± 0.00^a^	0.05 ± 0.02^a^	0.05 ± 0.00^a^	0.05 ± 0.00^a^	0.23 ± 0.01^a^	0.22 ± 0.01^a^
Stearic acid	2.45 ± 0.01^a^	2.65 ± 0.05^a^	2.50 ± 0.00^a^	3.07 ± 0.06^b^	6.07 ± 0.02^c^	5.99 ± 0.04^d^
Arachidic acid	0.39 ± 0.00^a^	0.41 ± 0.02^a^	0.40 ± 0.00^a^	0.42 ± 0.01^a^	0.86 ± 0.01^b^	0.41 ± 0.00^a^
Palmitoleic acid	0.10 ± 0.00^a^	0.10 ± 0.00^a^	0.09 ± 0.00^a^	0.10 ± 0.00^a^	1.09 ± 0.01^b^	1.06 ± 0.00^b^
Heptadecenoic acid	0.02 ± 0.00^a^	0.03 ± 0.00^a^	0.02 ± 0.00^a^	0.01 ± 0.00^a^	0.30 ± 0.01^b^	0.26 ± 0.01^ab^
Oleic acid	11.84 ± 0.31^a^	12.98 ± 0.33^b^	11.35 ± 0.08^c^	9.05 ± 0.00^d^	23.09 ± 0.04^e^	22.90 ± 0.00^e^
Eicosenoic acid	0.23 ± 0.00^a^	0.47 ± 0.00^a^	0.22 ± 0.00^a^	0.23 ± 0.00^a^	2.98 ± 0.00^b^	2.23 ± 0.00^c^
Linoleic acid	77.63 ± 0.10^a^	76.09 ± 0.29^b^	78.20 ± 0.07^c^	80.26 ± 0.16^d^	40.45 ± 0.17^e^	40.63 ± 0.06^e^
Linolenic acid	0.12 ± 0.00^a^	0.16 ± 0.00^a^	0.09 ± 0.00^a^	0.12 ± 0.00^a^	1.53 ± 0.00^b^	1.13 ± 0.00^c^

*Note:* The values of compounds are expressed as mean ± standard deviation. Values within each row with different superscript letters (a–e) are significantly different at *p* ≤ 0.05.

Abbreviations: SAM, Ait melloul safflower oil extracted by maceration; SAP, Ait melloul safflower oil extracted by cold press; SAS, Ait melloul safflower oil extracted by Soxhlet; SGM, Guelmim safflower oil extracted by maceration; SGP, Guelmim safflower oil extracted by cold press; SGS, Guelmim safflower oil extracted by Soxhlet.

The fatty acid profile varied depending on the cultivar and extraction method (Table 2). A total of eleven fatty acids were identified in both safflower varieties, with unsaturated fatty acids (UFA), particularly polyunsaturated fatty acids (PUFA), being predominant. Linoleic acid was the primary fatty acid detected, while palmitic and stearic acids were the main saturated fatty acids (SFA). Fatty acid composition is critical in defining the nutritional characteristics of vegetable oils (Hajib et al. 2018). Table 2 illustrates the fatty acid profile of oils extracted mechanically, as well as through Soxhlet and maceration, from safflower seeds from Ait melloul and Guelmim. The predominant fatty acids in all oils were palmitic, stearic, oleic, and linoleic acids, consistent with findings from earlier studies (Tonguc et al. 2023; Shirvani et al. 2016). Arachidic and eicosenoic acids were also present in small amounts, corroborating previous reports on safflower.

A significant difference in fatty acid content was observed between the seed origins. Ait melloul seed oils were higher in palmitic and linoleic acids during cold pressing, while Guelmim seed oils were richer in stearic and oleic acids. The extraction method also altered fatty acid percentages. Soxhlet extraction yielded oils with higher linoleic acid (78.20% and 80.25%) and lower oleic (11.34% and 9.04%), stearic (2.49% and 3.06%), and palmitic acids (6.94% and 6.41%).

Previous studies have highlighted the impact of extraction methods on safflower seed phytochemical profiles. Deviren and Aydın (2023) found that soxhlet extraction produced the highest oil yield (32.49%), while Song et al. (2023) demonstrated Soxhlet's efficiency in terms of oil yield, finding no significant difference in fatty acid composition between Soxhlet and cold pressing. However, Conte et al. (2016) reported significant differences in both extraction methods and conditions.

### 3.3 Qualitative, Nutritional, and Metabolic Indexes

Oils and fats significantly influence food's taste, flavor and quality, and there is a growing focus on natural products due to rising health awareness. The nutritional and metabolic indices of safflower cultivars (Ait melloul and Guelmim) were calculated based on fatty acid (FA) composition (Table 3). Significant differences were observed in the indices, each providing unique insights based on context.

**TABLE 3:** Nutritional and metabolic indices of safflower oils from Ait Melloul and Guelmim regions based on different extraction techniques.

	SAP	SGP	SAS	SGS	SAM	SGM
*Qualitative indexes*						
SFA	9.989 ± 0.247^a^	10.067 ± 0.094^a^	10.010 ± 0.040^a^	10.142 ± 0.092^a^	25.736 ± 0.339^b^	24.078 ± 0.048^c^
UFA	89.94 ± 0.416^a^	89.832 ± 0.038^a^	89.968 ± 0.154^a^	89.769 ± 0.165^a^	69.446 ± 0.110^b^	68.225 ± 0.08^c^
MUFA	12.192 ± 0.313^a^	13.587 ± 0.328^b^	11.675 ± 0.088^a^	9.389 ± 0.001^c^	27.463 ± 0.061^d^	26.460 ± 0.016^e^
PUFA	77.748 ± 0.103^a^	76.245 ± 0.290^b^	78.293 ± 0.066^a^	80.380 ± 0.164^c^	41.983 ± 0.171^d^	41.765 ± 0.064^d^
PUFA/SFA	7.786 ± 0.203^a^	7.574 ± 0.1^a^	7.822 ± 0.025^a^	7.926 ± 0.056^a^	1.631 ± 0.028^b^	1.735 ± 0.006^b^
Cox	8.140 ± 0.014^a^	8.001 ± 0.026^a^	8.188 ± 0.008^a^	8.383 ± 0.017^a^	4.728 ± 0.017^b^	4.659 ± 0.007^b^
OS	3517.34 ± 3.67^a^	3453.24 ± 8.93^b^	3539.92 ± 3.001^c^	3633.19 ± 7.38^d^	2000.95 ± 7.48^e^	1968.14 ± 2.88^f^
PI	78.162 ± 0.115^a^	76.727 ± 0.280^b^	78.669 ± 0.067^a^	80.728 ± 0.164^c^	44.092 ± 0.167^d^	43.470 ± 0.064^d^
UI	167.806 ± 0.523^a^	166.234 ± 0.25^b^	168.353 ± 0.219^a^	170.271 ± 0.330^c^	112.961 ± 0.279^d^	111.122 ± 0.144^e^
*Nutritional indexes*						
IA	0.084 ± 0.003^a^	0.0831 ± 0.003^a^	0.0826 ± 0.001^a^	0.079 ± 0.002^a^	0.390 ± 0.015^a^	0.350 ± 0.007^a^
IT	0.211 ± 0.006^a^	0.212 ± 0.001^a^	0.211 ± 0.001^a^	0.214 ± 0.001^a^	0.639 ± 0.010^a^	0.634 ± 0.002^a^
h/H	12.612 ± 0.487^a^	12.811 ± 0.016^a^	12.688 ± 0.05^a^	13.539 ± 0.013^b^	3.502 ± 0.068^c^	3.703 ± 0.024^c^
HPI	11.922 ± 0.471^a^	12.023 ± 0.068^a^	12.093 ± 0.03^a^	12.512 ± 0.053^a^	2.567 ± 0.096^b^	2.860 ± 0.054^b^
*Metabolic indexes*						
Elongase	35.186 ± 1.105^a^	38.920 ± 0.708^b^	35.966 ± 0.220^a^	47.794 ± 0.918^c^	38.534 ± 0.125^d^	39.049 ± 0.200^d^
Desaturase	82.859 ± 0.401^a^	83.073 ± 0.098^a^	81.966 ± 0.107^b^	74.689 ± 0.392^c^	79.183 ± 0.03^d^	79.276 ± 0.10^d^
ODR	86.787 ± 0.282^a^	85.451 ± 0.36^a^	87.344 ± 0.071^a^	89.883 ± 0.019^a^	64.515 ± 0.131^b^	64.584 ± 0.034^b^
LDR	0.151 ± 0.005^a^	0.206 ± 0.003^a^	0.118 ± 0.002^a^	0.152 ± 0.002^a^	3.649 ± 0.022^a^	2.710 ± 0.004^a^

*Note:* The values of compounds are expressed as mean ± standard deviation. Values within each row with different superscript letters (a–f) are significantly different at *p* ≤ 0.05.

Abbreviations: Cox, oxidizability; h/H, hypocholesterolemic FA/hypercholesterolemic FA ratio; HPI, health promoting index; IA, atherogenicity index; IT, thrombogenicity index; LDR, Linoleic desaturation ratio; ODR, Oleic desaturation ratio; OS, oxidative stability; PI, peroxidability; PUFA/SFA, Polyunsaturated FA/saturated FA ratio; SAM, Ait melloul safflower oil extracted by maceration; SAP, Ait melloul safflower oil extracted by cold press; SAS, Ait melloul safflower oil extracted by Soxhlet; SFA, saturated fatty acid; SGM, Guelmim safflower oil extracted by maceration; SGP, Guelmim safflower oil extracted by cold press; SGS, Guelmim safflower oil extracted by Soxhlet; UFA, unsaturated fatty acid; UI, unsaturation index.

Saturated fatty acid (SFA) content ranged from 9.99% to 25.74%, with the highest values from maceration extraction. This phenomenon is likely attributable to light exposure, which has been observed to lead to a loss of unsaturated fatty acids (UFA) and subsequent increase in the percentage of SFA (Jurić et al. 2022). Additionally, studies have indicated that SFAs can enhance the flexibility of cancer cells when they are administered exogenously, while replacing SFAs with UFA or proteins has been associated with improvements in lipid profiles, including reductions in atherogenic cholesterol markers (Jacobson et al. 2015). In alignment with the Food and Agriculture Organization (FAO) and the World Health Organization (WHO), polyunsaturated fats (PUFAs) are essential for cardiovascular disease prevention (Lise Halvorsen and Blomhoff 2011). Safflower seed oils from both regions exhibited high levels of UFAs (68.225%–89.968%), with PUFAs dominating. These differences were influenced by the area of origin and extraction method, ranging from 41.765% to 80.380%. Balanced PUFA/SFA ratios are vital for preventing cardiovascular diseases (CVD), autoimmune diseases, neurodegenerative conditions, and for improving mental health and cognitive functions (Giles et al. 2019). Since the human body cannot synthesize sufficient amounts of these essential FAs, maintaining a balanced dietary intake of PUFAs and SFAs is critical for optimal cholesterol levels (Plaha et al. 2023).

In this study, the PUFA/SFA ratio varied between 1.63 and 7.92, with all values exceeding 0.45, indicating the potential health benefits of safflower oil. While PUFAs offer cardiovascular protection, they are prone to peroxidation, affecting oil flavor and aroma. Lipid oxidation reduces the oil's nutritional value by destroying essential FAs, negatively impacting human health (Tao 2015). Therefore, assessing an oil's oxidation status and stability is essential for evaluating its nutritional quality.

To predict safflower oil oxidizability, theoretical oxidizability indices (Cox, OS, PI) were calculated (Table 3). While there was no significant difference in Cox values between cultivars, extraction methods revealed notable differences. Soxhlet‐extracted oils were more prone to oxidation (SGS: 8.38%, SAS: 8.18%) due to heat exposure. These values are comparable to *Colliguaya integerrima* (9.35%) but higher than *Olea europaea* (1.40%–2.85%), *Moringa oleifera* (0.82%), *Pennisetum glaucum* (3.83%–5.46%), *Opuntia ficus‐indica* (6.50%), and *Cynara cardunculus* (7.57%) oils (Abril et al. 2019; Moghaddam et al. 2012; Salama et al. 2020; El Kourchi, Belhoussaıne, Elhrech, et al. 2024). Oils with lower Cox and higher OS values are more resistant to oxidation (Plaha et al. 2023). Maceration‐extracted oils showed moderate oxidative stability and lower Cox values.

The peroxidability index (PI) revealed significant differences in Guelmim oils, where maceration‐extracted oils had the lowest PI (43.47%) compared to Soxhlet (80.72%) and cold‐pressed oils (76.72%). A low PI value indicates a longer shelf life and resistance to self‐oxidation, whereas a higher PI provides enhanced protection against coronary heart disease. Safflower oils with high PI values generally have higher PUFA levels, crucial for health but susceptible to oxidative destruction, which diminishes their nutritional quality and sensory properties (Wang et al. 2020).

The degree of unsaturation (UI) was calculated to assess the unsaturation weight of each UFA. Guelmim oils exhibited higher unsaturation levels, particularly in Soxhlet‐extracted oils. Environmental factors, such as heavy metal contamination, pesticide residues, and insect infestations, can significantly impact the safety and quality of vegetable oils during cultivation, processing, and storage (Zhou, Lai, et al. 2020).

The atherogenicity index (AI) ranged from 0.079 to 0.390, lower than in *Helianthus annus* (0.09) and *Linum usitatisimum* oils (0.09–0.23), but higher than in *Camelina sativa* (0.06–0.07) (Plaha et al. 2023; Filip et al. 2011; Ratusz et al. 2018). A low AI value is desirable for a heart‐healthy diet. The thrombogenicity index (TI), which assesses the balance between pro‐ and anti‐thrombogenic fatty acids, ranged from 0.211 to 0.639, significantly higher than in *Linum usitatisimum* (0.04–0.26), *Camelina sativa* (0.1), and *Lupinus albus* (0.13–0.18) oils (Plaha et al. 2023; Ratusz et al. 2018; Calabrò et al. 2015). A diet rich in low AI and TI foods is linked with more favorable lipid profiles and may contribute to improved cardiovascular health.

The hypocholesterolemic/hypercholesterolemic (h:H) ratio, which characterizes the balance between these fatty acids, ranged from 3.5 to 13.6, with Guelmim oils showing the highest value. These values are comparable to *Camelina sativa* oil and lower than in *Helianthus annus* (Machate et al. 2022). Safflower oil's high (h:H) ratio suggests a desirable balance between hypocholesterolemic PUFAs and hypercholesterolemic FAs, offering positive health effects.

The Health Promotion Index (HPI) ranged from 2.56 to 12.51, with no significant differences between cultivars, although Guelmim oils exhibited the highest index. A high HPI value suggests enhanced nutritional quality.

Lastly, four ratios were calculated to assess fatty acid biosynthesis. Elongation converts palmitic to stearic acid, and desaturation adds double bonds to form palmitoleic and oleic acids. Elongation proportions ranged from 35.18% to 47.79%, with considerable variation between cultivars. High desaturase activity was evident in pressed oils, with higher C18:1 level relative to C18:0, consistent with previous studies (Dal Bosco et al. 2022; Laborde et al. 2001). Oleic desaturation (ODR) and linoleic desaturation (LDR) ratios further highlighted the efficiency of these pathways, with high ODR and low LDR values recorded, particularly in Ait melloul oils. These findings underscore the effectiveness of the biosynthetic pathway for oleic and linoleic acids.

### 3.4 Physicochemical Parameters

**FIGURE 1:**
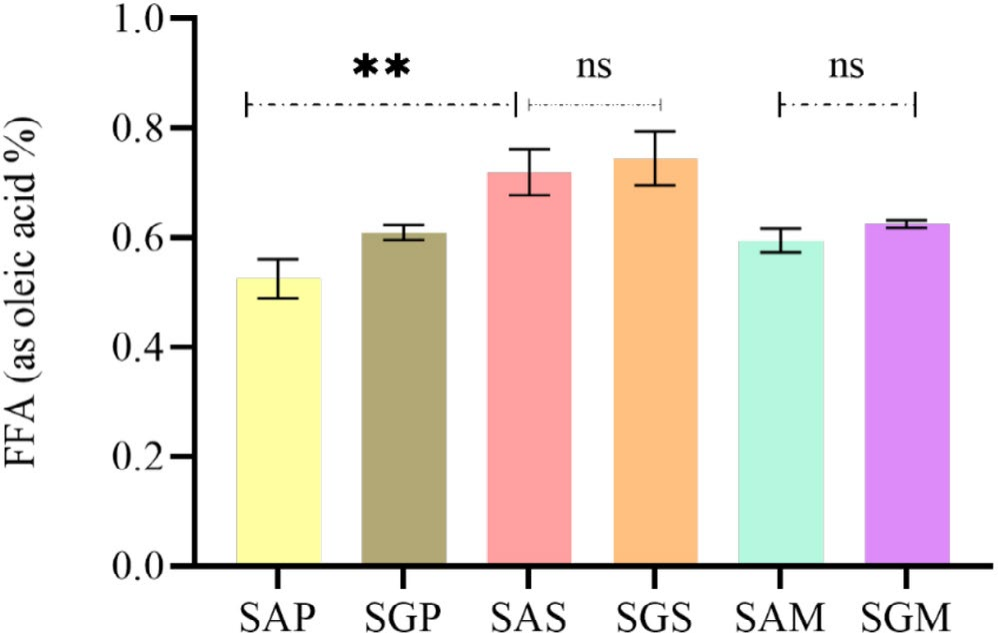
Free fatty acid (FFA) (as oleic acid %) content of safflower oils extracted from two cultivars using different methods (Soxhlet, cold pressing, and maceration). The values of compounds are expressed as mean ± standard deviation. Values with ** are significantly different at *p* ≤ 0.05 and those with ns are not significantly different. Abbreviations are: SAP, Ait melloul safflower oil extracted by cold press; SGP, Guelmim safflower oil extracted by cold press; SAS, Ait melloul safflower oil extracted by Soxhlet; SGS, Guelmim safflower oil extracted by Soxhlet; SAM, Ait melloul safflower oil extracted by maceration; SGM, Guelmim safflower oil extracted by maceration.

**FIGURE 2:**
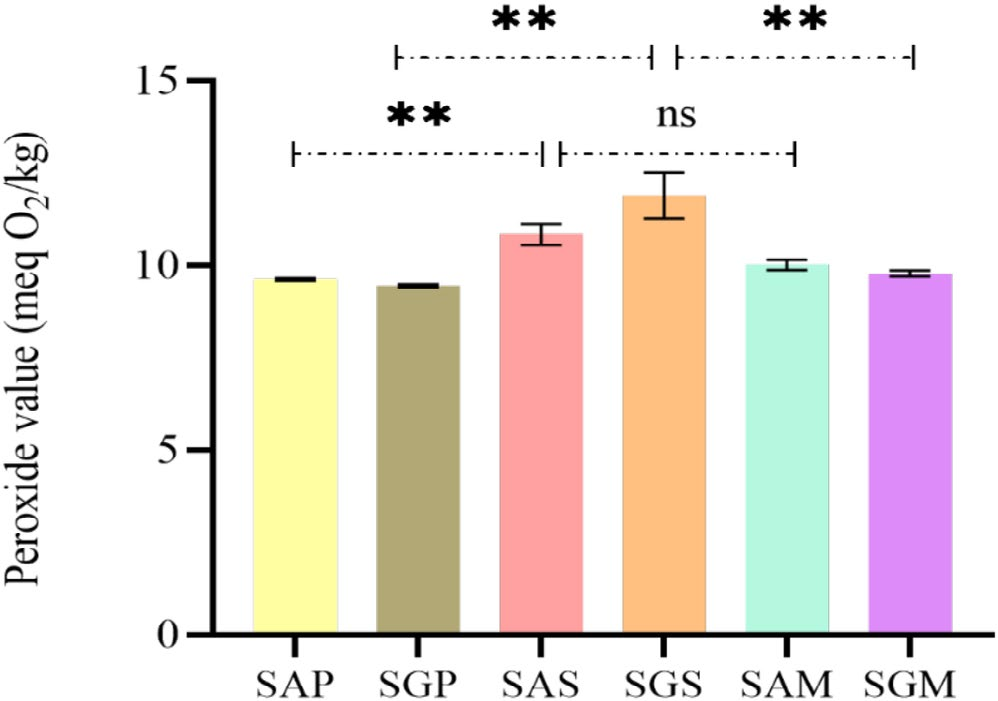
Peroxide value (PV) (meq O_2_/kg) of safflower oils extracted from two cultivars. The values of compounds are expressed as mean ± standard deviation. Values with ** are significantly different at *p* ≤ 0.05 and those with ns are not significantly different. Abbreviations are: SAP, Ait melloul safflower oil extracted by cold press; SGP, Guelmim safflower oil extracted by cold press; SAS, Ait melloul safflower oil extracted by Soxhlet; SGS, Guelmim safflower oil extracted by Soxhlet; SAM, Ait melloul safflower oil extracted by maceration; SGM, Guelmim safflower oil extracted by maceration.

**FIGURE 3:**
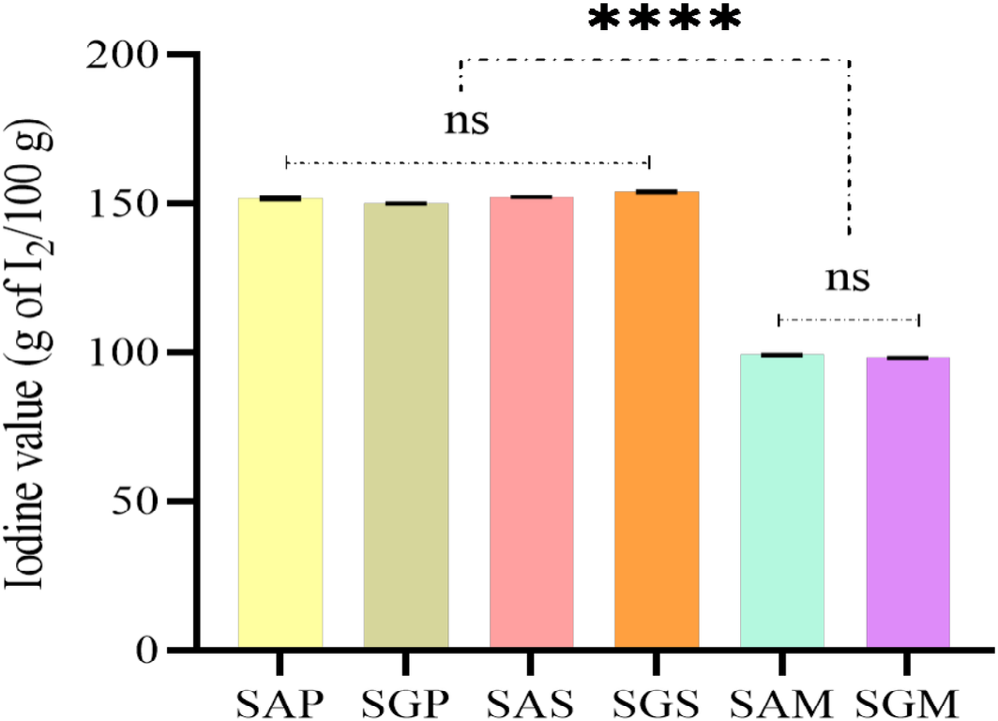
Iodine value (IV) (g I_2_/100 g) representing unsaturation levels in safflower oils. The values of compounds are expressed as mean ± standard deviation. Values with **** are significantly different at *p* ≤ 0.0001 and those with ns are not significantly different. Abbreviations are: SAP, Ait Melloul safflower oil extracted by cold press; SGP, Guelmim safflower oil extracted by cold press; SAS, Ait Melloul safflower oil extracted by Soxhlet; SGS, Guelmim safflower oil extracted by Soxhlet; SAM, Ait Melloul safflower oil extracted by maceration; SGM, Guelmim safflower oil extracted by maceration.

**FIGURE 4:**
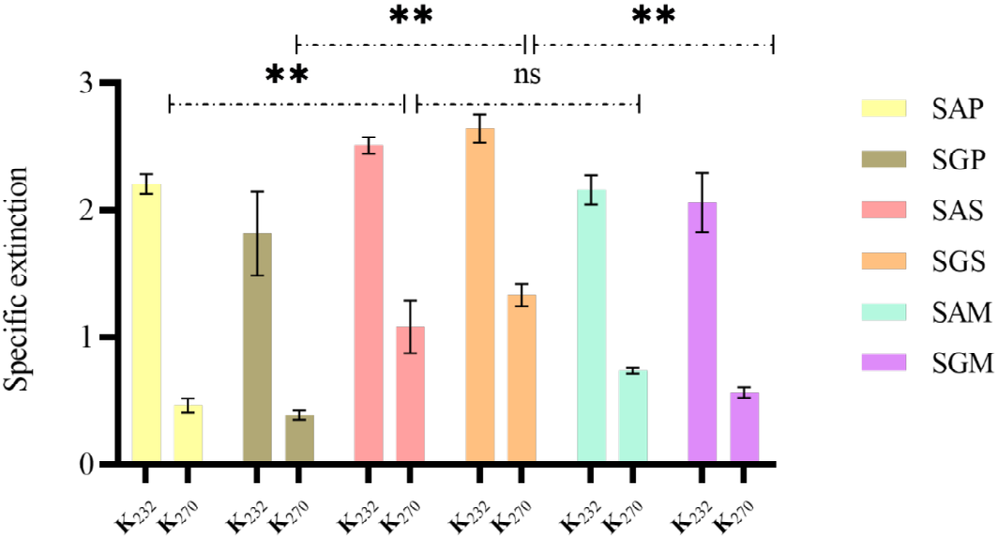
Specific extinction coefficients (K_232_ and K_270_) for primary and secondary oxidation products in safflower oils. The values of compounds are expressed as mean ± standard deviation. Values with ** are significantly different at *p* ≤ 0.05 and those with ns are not significantly different. Abbreviations are: SAP, Ait Melloul safflower oil extracted by cold press; SGP, Guelmim safflower oil extracted by cold press; SAS, Ait Melloul safflower oil extracted by Soxhlet; SGS, Guelmim safflower oil extracted by Soxhlet; SAM, Ait Melloul safflower oil extracted by maceration; SGM, Guelmim safflower oil extracted by maceration.

**FIGURE 5:**
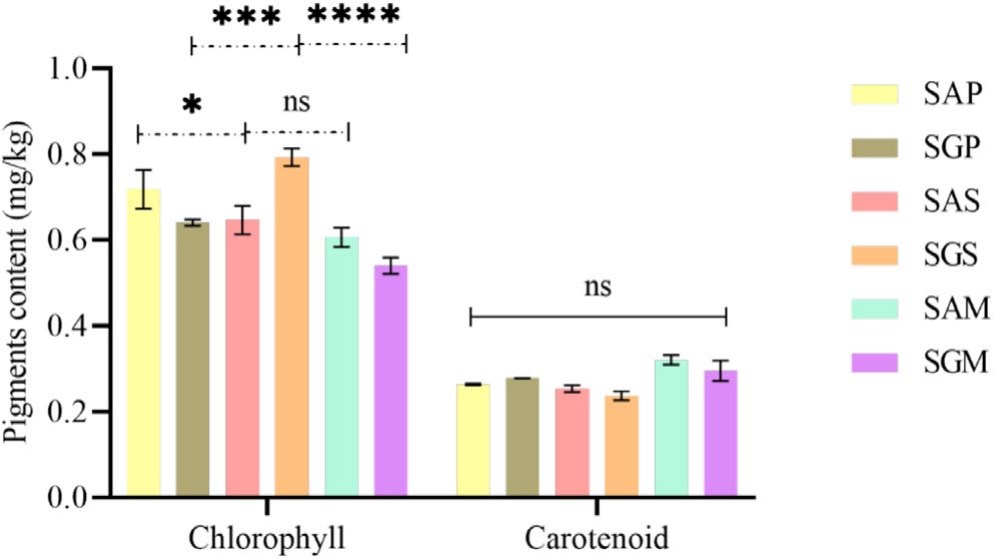
Pigment content (chlorophyll and carotenoid) in safflower oils. The values of compounds are expressed as mean ± standard deviation. Values with **** are significantly different at *p* ≤ 0.0001, *** are significantly different at *p* ≤ 0.0006, and those with ns are not significantly different. Abbreviations are: SAP, Ait Melloul safflower oil extracted by cold press; SGP, Guelmim safflower oil extracted by cold press; SAS, Ait Melloul safflower oil extracted by Soxhlet; SGS, Guelmim safflower oil extracted by Soxhlet; SAM, Ait Melloul safflower oil extracted by maceration; SGM, Guelmim safflower oil extracted by maceration.

The qualitative nutritional and metabolic parameters of safflower lipids have positioned them as key sources of techno‐functional benefits, though oil quality can degrade during processing, leading to hazardous substances such as hydroperoxides and oxidized sterols. Thus, the quality of these oils was evaluated by assessing free fatty acid (FFA) content, peroxide value (PV), unsaturation levels, oxidation products (primary and secondary) and pigments (chlorophyll and carotenoids) (Figures 1, 2, 3, 4, 5).

The FFA content revealed a significant difference between extraction methods, particularly between press and Soxhlet for both locations (Figure 1). Oils extracted without solvents contained lower FFA percentages, with values ranging from 0.52% to 0.72% for SG and 0.61% to 0.74% for SA. Although these results are higher than those reported for Egyptian cultivars and lower than those for Ethiopian cultivars, they align with findings for Turkish cultivars (Aydeniz et al. 2014; Cosge Senkal et al. 2016; Al Surmi et al. 2015). The higher acidity observed in Soxhlet extraction suggests temperature influences FA release through hydrolysis. Short hydrocarbon chains in FFA also make them more easily extracted by solvents.

Peroxide values varied significantly between extraction methods for SG (9.45–11.89 meq O_2_/kg), while SA showed a difference between Soxhlet and cold pressing (9.62–10.84 meq O_2_/kg) (Figure 2). These values are higher than those found in Tunisia, Turkey, Egypt, Ethiopia, and Iran (Aydeniz et al. 2014; Cosge Senkal et al. 2016; Al Surmi et al. 2015; Ahmadzadeh et al. 2014; Khémiri et al. 2020). Despite the absence of any indication of oil degradation, the extraction method proved to have a significant impact on the peroxide content. Specifically, Soxhlet oils demonstrated higher values as a consequence of temperature and solvent exposure (Özcan et al. 2019; Wang et al. 2022). Importantly, both cultivars extracted without solvents scored below 15 meq O_2_/kg, aligning with the CODEX ALIMENTARIUS standard for cold pressed fat and oils (Codex Alimentarius 2023). The iodine index, representing unsaturation levels, ranged from 98.232 to 154.069 g I_2_/100g for SG and 99.232 to 152.287 g I_2_/100g for SA (Figure 3). Higher unsaturation levels in Soxhlet‐extracted oils align with the fatty acid profile and suggest increased susceptibility to oxidation (Boujemaa et al. 2020; Serjouie et al. 2010). Comparatively, the index was lower than that reported for Iranian safflower oils (Ahmadzadeh et al. 2014).

Primary and secondary oxidation products were measured through extinction coefficients (K_232_ and K_270_), with significant differences depending on the extraction method (Figure 4). SG oils showed K_232_ values between 1.81 and 2.64, while SA oils ranged from 2.15 to 2.50. K_270_ values revealed Soxhlet extraction increased secondary oxidation products, particularly in Guelmim seed oils, correlating with the oxidation markers. Conjugated triene formation was resistant in these oils.

Chlorophyll and carotenoid pigments were also measured, with chlorophyll acting as a pro‐oxidant and carotenoids as antioxidants (Figure 5). SG had higher chlorophyll content (0.54–0.79 mg/kg) in Soxhlet‐extracted oils, consistent with peroxide and extinction coefficient values, summarizing the effect of oxidation. Carotenoid content was lower than chlorophyll in all methods, with Soxhlet extraction yielding the lowest values. Pigment content is influenced by plant cultivation and seed maturity, and carotenoids are known for their cancer‐preventive properties (Maoka 2020).

### 3.5 Tocopherols Content

Safflower oil's nutritional profile, especially its high PUFA content, alongside elevated peroxide, specific extinction and iodine values, makes it susceptible to peroxidation. Reactive oxygen species trigger lipid peroxidation in PUFAs. Various factors, such as heat, light, metallic ions, and oxygen exposure during oil production, exacerbate this auto‐oxidation (Islam et al. 2023). Tocopherols, known antioxidants, protect PUFAs from oxidation, with their efficacy dependent on total tocopherol (TT) content relative to PUFA levels.

In this study, tocopherol content was extracted using HPLC, and the results are shown in Table 4. TT and its isoforms ranged from 596.29 to 860.21 mg/kg, depending on the cultivar and extraction method. Solvent‐free extraction yielded the highest TT content, consistent with Fernández‐Cuesta et al. (2014), who found that cold‐pressed oils generally have higher TT concentrations compared to solvent‐extracted oils. Ait melloul seed oils exhibited higher TT values regardless of the extraction technique.

**TABLE 4:** Tocopherol content (α‐tocopherol and β‐tocopherol) (mg/kg) in safflower oils from two regions, extracted using various methods.

	SAP	SGP	SAS	SGS	SAM	SGM
α‐t	837.61 ± 0.22^a^	831.18 ± 0.03^b^	729.88 ± 0.17^c^	631.47 ± 0.06^d^	589.72 ± 0.21^e^	580.67 ± 0.27^f^
β‐t	18.10 ± 0.07^a^	21.08 ± 0.02^b^	40.37 ± 0.12^c^	10.34 ± 0.09^d^	62.58 ± 0.28^e^	3.62 ± 0.22^f^
TT	860.21 ± 0.15^a^	855.3 ± 0.12^b^	770.25 ± 0.04^c^	648.17 ± 0.05^d^	652.3 ± 0.42^e^	596.29 ± 0.07^f^

*Note:* The values of compounds are expressed as mean ± standard deviation. Values within each row with different superscript letters (a–f) are significantly different at *p* ≤ 0.05.

Abbreviations: SAM, Ait melloul safflower oil extracted by maceration; SAP, Ait melloul safflower oil extracted by cold press; SAS, Ait melloul safflower oil extracted by Soxhlet; SGM, Guelmim safflower oil extracted by maceration; SGP, Guelmim safflower oil extracted by cold press; SGS, Guelmim safflower oil extracted by Soxhlet; TT, Total tocopherol; α‐t, α‐tocopherol; β‐t, β‐tocopherol.

The study identified only two tocopherol isoforms: α‐tocopherol and β‐tocopherol. Several reports have stated that safflower oil can contain γ‐tocopherol, but usually in trace or low concentrations (0.0002%) (Ergönül and Özbek 2020; Matthaus et al. 2015). Matthaus et al. (2015) found safflower oil contains three types of tocopherols (α, β, and γ). α‐tocopherol was the major isomer, followed by β‐ and γ‐tocopherol, respectively. In addition, four cultivars of safflower were found to be completely devoid of γ‐tocopherol. The presence of this component is influenced by geographical location, processing of oil and storage conditions, possibly explaining its absence in our study. Furthermore, Günç Ergönül and Aksoylu Özbek (2018) found two isomers of tocopherols (α and γ) in five varieties of safflower with a complete absence of β and δ‐tocopherols. However, α‐tocopherol was the dominant tocopherol, accounting for 94%–96% of total tocopherols in safflower seed oil, with higher levels in oil obtained by cold pressing compared to oils obtained by solvent extraction (Aydeniz et al. 2014). A significant difference was observed in α‐tocopherol content between SA (ranging from 589.72 to 837.61 mg/kg) and SG (580.66 to 831.18 mg/kg), with SA oils containing higher amounts of α‐tocopherol than SG oils, irrespective of the extraction method. Cold pressing proved most effective in extracting tocopherols, followed by Soxhlet and maceration. These findings align with previous studies reporting significant α‐tocopherol content in safflower oil (Matthaus et al. 2015; Günç Ergönül and Aksoylu Özbek 2018; Ben Moumen et al. 2015; Lee et al. 2004).

In contrast, β‐tocopherol content showed a significant difference between cultivars and extraction methods. For SA, β‐tocopherol ranged from 18.09 to 62.57 mg/kg, while for SG, it ranged from 3.61 to 21.08 mg/kg. Previous studies also confirmed β‐tocopherol as the second most abundant tocopherol in safflower oil (Matthaus et al. 2015; Ben Moumen et al. 2015; Lee et al. 2004), although some reports, such as Günç Ergönül and Aksoylu Özbek (2018), noted the absence of β‐tocopherol in Turkish safflower varieties.

### 3.6 Correlation Analysis

Pearson correlation analysis was conducted based on the chemical composition (FAs and tocopherols), qualitative, nutritional, and metabolic indices, and physicochemical properties of safflower oil (Table 5). Safflower oil from *Carthamus tinctorius* displayed a significant positive correlation between linoleic acid and key indices derived from the FAs profile, such as PUFA/SFA, UI, Cox, and physicochemical indices like iodine value (IV) and absorbance at 670 nm (A670). A weak positive correlation was observed between linoleic acid and peroxide value (PV) and specific extinction coefficients (K_232_, K_270_). The results suggest that safflower oil is prone to autooxidation due to its high PUFA and chlorophyll content (prooxidants) as well as its unsaturation levels (IV, UI). However, the high concentration of total tocopherols (TT) provides antioxidative protection, reducing the peroxidation risk, as evidenced by the low correlation between linoleic acid and the peroxidability indices (PV, K_232_, K_270_), contributing to the oil's stability. Notably, only α‐tocopherol (α‐T) showed a highly positive correlation with C18:2, while β‐tocopherol (β‐T) exhibited a significant negative correlation with C18:2. Tocopherols, particularly α‐T, play a protective role against the oxidative damage of highly unsaturated fatty acids, and α‐T's membrane‐stabilizing effects resemble cholesterol, stabilizing PUFA‐rich domains in cellular membranes (Atkinson et al. 2010). Thus, oil quality is influenced by both its chemical composition and the balance between pro‐oxidants and antioxidants. From a nutritional perspective, safflower oil showed a strong positive correlation between the hypocholesterolemic/hypercholesterolemic (h:H) ratio and linoleic acid concentration, as well as with FA quality indices (*r* = 0.998), a moderate positive correlation with TT content (*r* = 0.677), and weaker correlations with PV (*r* = 0.356), K_232_ (*r* = 0.348), and K_270_ (*r* = 0.271). FAs can play either a positive or negative role in chronic disease prevention, depending on their degree of saturation (Shahidi and Zhong 2010). SFAs, for instance, have been linked to coronary heart disease, prompting health authorities to recommend limiting SFA intake to no more than 10% of total energy for healthy individuals and 5%–6% for those with dyslipidemia or atherosclerosis (Pearson et al. 2021). A negative correlation between the (h:H) ratio and palmitic acid further supports the notion that reducing C16:0 levels can increase the presence of cholesterol‐lowering FAs. PUFAs, particularly omega‐6 (linoleic acid) and omega‐3 (linolenic acid), have been associated with potential benefits for cardiovascular health and are essential fatty acids (EFAs) because the body cannot synthesize them. Dietary intake of EFAs has been linked to the prevention of diseases such as renal hypertension, cardiovascular disease, type 2 diabetes, dermatitis, and arthritis. The high linoleic acid content in safflower oil has been shown to reduce total cholesterol and low‐density lipoprotein (LDL) cholesterol when consumed in moderation (Froyen and Burns‐Whitmore 2020). This functionality is also characteristic of phytosterols, which represent natural steroids widely present in various parts of plants and are an important component of plant cell membranes. In addition, safflower exhibits a notably elevated total phytosterol content, which is subject to variation due to variety and environmental influences. According to extant literature, the most abundant phytosterol in safflower oil is β‐sitosterol (Ben Moumen et al. 2015). Indeed, the active ingredient β‐sitosterol has been identified as a critical component in the development of anti‐atherosclerotic treatment strategies. In experimental models involving animals, the administration of a diet comprising 2% combined phytosterols has been demonstrated to impede the progression and dissemination of cancer cells, as well as the emergence of colon, breast, and prostate cancer in animals exposed to diverse carcinogens (Khan et al. 2022).

**TABLE 5:** Pearson correlation between fatty acid profile, tocopherol content, nutritional indices and physicochemical properties of safflower oils.

	C16: 0	C18:1	C18:2	C18:3	PUFA/SFA	UI	Cox	h/H	ODR	LDR	IV	PV	K_232_	K_270_	A_670_	A_470_	TT	αT	βT
C16:0	**1**																		
C18:1	0.983	**1**																	
C18:2	−0.998	−0.990	**1**																
C18:3	0.983	0.962	−0.979	**1**															
PUFA/SFA	−0.998	−0.984	0.999	−0.981	**1**														
UI	−0.998	−0.985	0.999	−0.975	0.999	**1**													
Cox	−0.997	−0.989	0.999	−0.975	0.999	0.999	**1**												
h/H	−0.999	−0.987	0.998	−0.979	0.998	0.998	0.998	**1**											
ODR	−0.995	−0.996	0.998	−0.974	0.995	0.996	0.998	0.997	**1**										
LDR	0.986	0.964	−0.981	0.999	−0.983	−0.977	−0.978	−0.982	−0.976	**1**									
IV	−0.998	−0.986	0.999	−0.977	0.999	0.999	0.999	0.998	0.996	−0.979	**1**								
PV	−0.332	−0.484	0.366	−0.298	0.333	0.346	0.366	0.356	0.412	−0.294	0.346	**1**							
K_232_	−0.330	−0.483	0.376	−0.313	0.348	0.358	0.376	0.348	0.417	−0.305	0.358	0.913	**1**						
K_270_	−0.248	−0.400	0.286	−0.212	0.255	0.269	0.287	0.271	0.330	−0.207	0.268	0.982	0.928	**1**					
A_670_	−0.753	−0.833	0.774	−0.681	0.755	0.768	0.778	0.769	0.800	−0.685	0.766	0.627	0.618	0.524	**1**				
A_470_	0.874	0.929	−0.884	0.899	−0.873	−0.869	−0.880	−0.879	−0.902	0.895	−0.872	−0.644	−0.644	−0.556	−0.782	**1**			
TT	−0.692	−0.570	0.675	−0.668	0.699	0.695	0.678	0.677	0.637	−0.674	0.694	−0.400	−0.296	−0.431	0.271	−0.293	**1**		
αT	−0.739	−0.618	0.718	−0.732	0.742	0.735	0.719	0.723	0.682	−0.738	0.734	−0.377	−0.293	−0.431	0.306	−0.373	0.987	**1**	
βT	0.290	0.284	−0.260	0.406	−0.260	−0.238	−0.248	−0.282	−0.268	0.401	−0.245	−0.057	0.066	0.090	−0.174	0.470	0.0293	−0.12	**1**

*Note:* Values in bold are different from 0 at significance level alpha = 0.05.

In addition to FAs, tocopherols and tocotrienols can reduce cholesterol production by inhibiting its biosynthesis in the liver (Rizvi et al. 2014). The positive correlation between the (h:H) ratio and TT content (*r* = 0.677) suggests a strong relationship between tocopherols and the prevention of hyperlipidemia. Furthermore, tocopherols have been shown to inhibit platelet aggregation, providing additional cardiovascular protection (Rizvi et al. 2014). EFAs are essential for human consumption, as humans cannot synthesize them. Long‐chain PUFAs are synthesized by desaturases that introduce additional unsaturation into existing PUFAs. These enzymes are found in plants, animals, and lower eukaryotes but not in mammals (Zemour et al. 2021). The desaturation pathways from linoleic to linolenic acid (LDR) and oleic to linoleic acid (ODR) were assessed. ODR had a strong positive correlation with C18:2 (*r* = 0.998), indicating high Δ‐12‐D desaturase activity and LDR showed a similar trend, with a correlation coefficient of *r* = 0.999 between LDR and C18:3. In plants, stearic acid is desaturated to oleic acid by Stearoyl‐CoA desaturase and C18:1 is further desaturated by Δ‐12‐D to linoleic acid and Δ‐15‐D to linolenic acid. These EFAs are then converted into gamma‐linoleic acid (GLA) and stearidonic acid (SA) by Δ‐6‐D, followed by elongation into Dihomo‐gamma linoleic acid (DGLA) and eicosatetraenoic acid (ETA). Further desaturation by Δ‐5‐D produces arachidonic acid (AA) and eicosapentaenoic acid (EPA) and subsequent elongation and desaturation yield docosapentaenoic acid (DPA) and docosahexaenoic acid (DHA), precursors to anti‐inflammatory docosanoids (Calder 2013).

In recent years, safflower, an emerging oilseed crop, has garnered significant attention due to the superior quality of its oil and its favorable agronomic characteristics, particularly its tolerance to drought and cold, rendering it well suited to Mediterranean climates (Abou Chehade et al. 2022). The significance attributed to safflower stems is predominantly attributable to the superior quality of the oil extracted from its seeds, with particular emphasis on the variability of its fatty acid content (Zemour et al. 2021). This variability is attributable to several factors that have prompted significant interest in scientific research initiatives in the Mediterranean region. In light of these considerations, a comparative study has been undertaken, encompassing both the diversity of Moroccan cultivars and the methodologies employed for extracting vegetable oils from this oilseed species. This initiative signifies a collaborative effort between the agri‐food and industrial sectors, with the objective of fostering the development and marketing of this species as an oilseed that possesses both nutritional and cosmetic benefits. From a nutritional perspective, safflower oil is notable for its high content of EFA, particularly linoleic (40.451%–80.258%) and oleic (9.047%–23.092%) acids. These components confer several beneficial properties, including hypocholesterolemic, antiatherogenic, and antithrombogenic effects, which collectively contribute to the primary prevention of atherosclerotic cardiovascular disease. Furthermore, safflower oil exhibited a robust positive correlation between linoleic acid, h/H ratio, total tocopherol, and α‐tocopherol content, thereby substantiating the nutritional value of this oleaginous species.

## 4 Conclusions

In recent years, there has been an increasing focus on healthy lifestyles, aiming for balanced, sustainable diets that ensure nutritional security and well‐being through optimized natural resources. This study evaluated the impact of cultivar variety and extraction methods on the phytochemical, nutritional and metabolic profile of *Carthamus tinctorius* seed oils. Moroccan safflower oil, rich in bioactive compounds, holds promise for food, pharmaceutical and cosmetic applications. While no major differences between cultivars were found, extraction methods significantly affected oil quality. Soxhlet extraction provided a high fat yield and linoleic acid amount but increased the oil's susceptibility to oxidation, as shown by indices like PV, K_232_ and K_270_. Nevertheless, extraction by maceration yielded higher concentrations of oleic, myristic and palmitic acid. Conversely, mechanical extraction produced elevated levels of total tocopherols, with α‐tocopherol exhibiting a particularly pronounced increase, which known for its ability to resist oxidative damage. Nutritionally, safflower oil's high content of essential fatty acids, especially linoleic (77.63%) and oleic acids (12.98%), confers beneficial hypocholesterolemic, antiatherogenic and antithrombogenic properties. Therefore, including *Carthamus tinctorius* seeds in the diet promotes a healthy and sustainable lifestyle.

## Author Contributions

**Filippo Maggi:** writing – review and editing, investigation. **Chaimae El Kourchi:** methodology, conceptualization, writing – original draft, investigation, data curation, formal analysis, resources. **Oumayma Belhoussaine:** conceptualization, methodology, visualization, writing – original draft, data curation. **Hicham Harhar:** conceptualization, supervision, writing – review and editing, methodology. **Rim Mohammed Ali:** methodology, data curation, writing – review and editing. **Mohammed Amakhmakh:** methodology, data curation. **Abdelhakim Bouyahya:** supervision, methodology, conceptualization, investigation, writing – review and editing, validation, project administration. **Mohamed Tabyaouı:** supervision, writing – review and editing, visualization. **Giovanni Caprioli:** formal analysis, writing – review and editing, investigation, validation. **Khalid M. Sumaily:** funding acquisition, investigation, writing – review and editing. **Agnese Santanatoglia:** writing – review and editing, investigation, validation.

## Conflicts of Interest

The authors declare no conflicts of interest.

## Data Availability

The data that support the findings of this study are available on request from the corresponding author. The data are not publicly available due to privacy or ethical restrictions.
